# Proteomic analysis reveals proteins and pathways associated with declined testosterone production in male obese mice after chronic high-altitude exposure

**DOI:** 10.3389/fendo.2022.1046901

**Published:** 2022-11-30

**Authors:** Shuqiong Wang, Youwen Wei, Caiyan Hu, Fang Liu

**Affiliations:** ^1^ Research Center for High Altitude Medicine, Qinghai University, Xining, China; ^2^ Key Laboratory of High Altitude Medicine, Ministry of Education, Xining, China; ^3^ Key Laboratory of Application and Foundation for High Altitude Medicine Research in Qinghai Province, Qinghai-Utah Joint Research Key Lab for High Altitude Medicine, Xining, China; ^4^ Department of Endocrinology, Qinghai Provincial People’s Hospital, Xining, China; ^5^ Department of Plague Prevention and Control, Qinghai Institute for Endemic Disease Prevention and Control, Xining, China; ^6^ Department of Laboratory Medicine, Baoding First Central Hospital, Baoding, China; ^7^ Department of Biochemistry, Medical College, Qinghai University, Xining, China

**Keywords:** testosterone, obese, hypoxia, testis, proteomics, oxidative stress

## Abstract

**Objective:**

Obesity is common in highland areas owing to lifestyle alterations. There are pieces of evidence to suggest that both obesity and hypoxia may promote oxidative stress, leading to hypogonadism in males. These findings indicate an increased risk of hypogonadism in obese males following hypoxia exposure. However, the mechanisms underlying the disease process remain unclear. The current study aims to explore the mechanism of testosterone production dysfunction in obese male mice exposed to a chronic high-altitude hypoxia environment.

**Methods:**

An obese male mouse model was generated by inducing obesity in mice via a high-fat diet for 14 weeks, and the obese mice were then exposed to a high-altitude hypoxia environment for 24 days. Sera and testicular tissues were collected to detect serum lipids, sex hormone level, and testicular oxidative stress indicators. Morphological examination was performed to assess pathological alterations in testicular tissues and suborganelles in leydig cells. Proteomic alterations in testicular tissues were investigated using quantitative proteomics in Obese/Control and Obese-Hypoxia/Obese groups.

**Results:**

The results showed that chronic high-altitude hypoxia exposure aggravated low testosterone production in obese male mice accompanied by increased testicular oxidative stress and histological damages. In total, 363 and 242 differentially expressed proteins (DEPs) were identified in the two comparison groups, Obese/Control and Obese-Hypoxia/Obese, respectively. Functional enrichment analysis demonstrated that several significant functional terms and pathways related to testosterone production were altered in the two comparison groups. These included cholesterol metabolism, steroid hormone biosynthesis, peroxisome proliferator-activated receptor (PPAR) signaling pathway, oxidative stress responses, as well as retinol metabolism. Finally, 10 representative DEPs were selected for parallel reaction monitoring verification. Among them, StAR, DHCR7, NSDHL, CYP51A1, FDPS, FDX1, CYP11A1, ALDH1A1, and GPX3 were confirmed to be downregulated in the two groups.

**Conclusions:**

Chronic hypoxia exposure could exacerbate low testosterone production in obese male mice by influencing the expression of key proteins involved in steroid hormone biosynthesis, cholesterol biosynthesis, oxidative stress responses and retinol metabolism.

## 1 Introduction

Obesity has become a global epidemic that can trigger a variety of related complications including male obesity secondary hypogonadism (1). Large scale epidemiological studies and small-scale population surveys have suggested that its prevalence may be as high as 45.0–57.5% (2, 3). This condition is frequently characterized by a low testosterone level that impairs male fertility, reproductive hormones, bone mineralization, fat metabolism, and muscle mass (1). Obesity is common in highland areas due to lifestyle alterations (4). Recently, increased obesity rates among urban residents exposed to high altitudes (5) and commuters under intermittent high-altitude exposure (4) have been reported. Subjects with higher body mass index (BMI) among highland settlers were more prone to exhibit bad responses to hypoxia (6). In addition, studies have found that plateau hypoxia has a certain effect on male reproductive hormones, and the intensity of the impact rises as altitude increases (7). These studies imply that chronic hypoxia exposure may increase the risk of hypogonadism in male obesity. Although the low testosterone level induced by male obesity can be reversed partly with weight loss (8), weight loss strategy for obese people in peculiar high-altitude environments is a double-edged sword and may increase the risk of hypoxemia (9, 10). Therefore, this study seeks to explore the mechanisms underlying how chronic hypobaric hypoxia exposure perturbs the testosterone production in male obesity. The results of this study can help ensure the physical and mental health of migrant workers and local residents in highland regions.

Oxidative stress (OS) in the testis plays a crucial role in male hypogonadism (11). Testicular OS can lead to low testosterone productivity, which is most likely attributed to the impairment of Leydig cells (LCs) function during this process (12). Previous studies have reported that OS is a crucial pathogenic factor in male hypogonadism induced by obesity (13). In addition, evidence suggests that hypoxia instigates the production and accumulation of reactive oxygen species (ROS) (14), which can result in hypogonadism and infertility (15). Both obesity and hypoxia are suggested to cause male hypogonadism *via* increased OS. However, the underlying molecular mechanism remains unknown.

LCs synthesize and secrete most of the testosterone (95%) in the testes to maintain normal reproductive and gonadal functions in males. Testosterone production relies on external (hypothalamic–pituitary–gonadal (HPG) axis) and internal factors (substrate cholesterol availability, trafficking, and steroidogenic enzyme activities) (16). In the case of internal factors, cholesterol is the main substrate for testosterone biosynthesis, which is derived from *de novo*–synthesized cholesterol (17), circulating cholesterol, and mobilized lipid droplets (LDs) (18). Once cholesterol is transported from cytosol to the internal mitochondrial membrane *via* its interaction with a large protein complex termed the steroidogenic acute regulatory protein (StAR), it is metabolized into pregnenolone by the cholesterol side-chain cleavage enzyme (CYP11A1) by receiving electrons from nicotinamide adenine dinucleotide phosphate (NADPH) *via* ferredoxin-1(FDX1), a redox partner protein that maintains the activity of CYP11A1 (19, 20). Subsequently, it is converted into androgens by several steroidogenic enzymes in the mitochondria and the endoplasmic reticulum (ER). Enzymes involved in testosterone biosynthesis include 3 beta-hydroxysteroid dehydrogenase/Delta 5-->4-isomerase type 1 (HSD3B1), steroid 17-alpha-hydroxylase/17,20 lyase (CYP17A1), and 17-beta-hydroxysteroid dehydrogenase type 3 (HSD17B3) (21, 22).

Mitochondria are the key sites of testicular steroid synthesis, while also being both the target and source of ROS. Low ROS levels are necessary for the regulation of normal gonadal and reproductive functions in men. ROS overproduction directly damages the mitochondrial respiratory chain complexes and disrupts the electron transfer, all of which result in the impairment of mitochondrial function and reduction of testosterone synthesis in LCs (23). This evidence indicates that mitochondria should be fully functional for testosterone synthesis in LCs (24). Previous studies have reported that OS and inflammation in the testis induced by obesity reduced the production of testosterone (25). The increase of mitochondrial substrate load in obesity often induces lipid peroxidation and destroys the antioxidant defense system, which further interferes with the capacity for steroid synthesis and cholesterol transport and synthesis, ultimately suppressing the level of testosterone synthesis (25, 26). In addition, the reduction in testosterone level has been reported to be associated with chronic and intermittent periods of hypoxia in rodents (27) and humans (28). Similar cases were found in patients suffering from hypoxemia due to obstructive sleep apnea (29). Most of these studies postulated that OS caused by excess ROS in the testis is one of the important mechanisms responsible for the decline of testosterone in chronic hypoxia (30, 31).

Therefore, diet-induced obese male mice exposed to a chronic hypoxia environment were used in this study to identify differentially expressed proteins (DEPs) in the testis using 4D label-free proteomic techniques in order to investigate the potential molecular mechanism that underlies testosterone production dysfunction triggered by chronic high-altitude hypoxia exposure. Additionally, serum hormone level and testicular OS-related indicators were detected, and the testicular tissue histopathology and the structures of the mitochondria in LCs were visualized. Eventually, the representative testicular DEPs related to testosterone production were validated through parallel reaction monitoring (PRM) targeted quantitation analysis. Until now, this study is the only one to report on testicular proteomic alterations in obese male mice exposed to a chronic hypoxia field environment. Thus, the findings in this study would make a novel contribution to the literature by exploring the impacts of chronic hypoxia on biological pathways or protein networks related to testosterone production under specific environmental conditions.

## 2 Materials and methods

### 2.1 Animals

#### 2.1.1 Overview

Six-week-old C57BL/6J male mice were purchased from Beijing Vital River Laboratory Animal Technology Co., Ltd (Shanghai branch; Certificate No.: 2017-0014, Shanghai, China) and housed under standard conditions: temperature, 22 ± 2°C; humidity, 53 ± 3%; and 12‐h light/12-h dark cycle. Food and water were provided ad libitum for adaptation.

#### 2.1.2 Ethics statement

The animal study was reviewed and approved by Animal Ethics Committee of Qinghai Provincial People’s Hospital affiliated to Qinghai University (No. 2021-39). All experimental procedures were performed in strict accordance with the National Institutes of Health Guide for the Care and Use of Laboratory Animals.

#### 2.1.3 Replication of diet-induced obese mouse model

After one week of adaptation feeding, seven-week-old C57BL/6J male mice were randomly selected and provided with either normal diet (ND) containing 15% fat (Beijing Keaoxieli Feed Co., Ltd., China) or high-fat diet (HFD) containing 60% fat (D12492, Research Diets, Inc, USA) for 14 weeks in Shanghai, China. Animals were housed under the following conditions: temperature, 22 ± 2°C; humidity, 53 ± 3%; and 12‐h light/12-h dark cycle. Each cage housed 3 mice with free access to food and water. The mice on HFD were considered to meet the criterion of obese mouse model when they consequently gained a body weight greater than 20% of that of ND mice (32). Body weight of all animals was acclimated once every two weeks.

#### 2.1.4 High-altitude hypoxia exposure in obese mice

Obese mice were randomly exposed simultaneously to either normoxic (Shanghai, China, altitude: 4.5 meters, 30°91′79″ N, 121°47′41″ E) or hypoxic conditions (The northeast of Qinghai province, China, altitude: 3810 meters, 36°17′18″ N, 100°53′48″ E) for 24 days while they continued to be fed with HFD. The factors selecting 24 days of high-altitude exposure include the general definition of acute and chronic hypoxia duration in most studies of the effects of hypoxia on animal reproduction, acclimatization at high altitudes for animals, the hidden risk of mortality in obese-hypoxia animals, and the potential for animal aging to interfere with study results. The obese mice that were randomly selected for hypoxic exposure were transported for three hours by plane and five hours by bus from normoxic (Shanghai, China; altitude: 4.5 m) to hypoxic conditions (northeast region of Qinghai province, China; altitude: 3810 m).They arrived at 7:00 p.m. and were housed under the following conditions: temperature, 20 ± 2°C; humidity, 45 ± 5%; and 12‐h light/12-h dark cycle. Each cage housed 3–4 mice with free access to food and water. Samples were collected on day 6, 15, and 24 following the exposure to normoxic or hypoxic conditions.

#### 2.1.5 Grouping methodology

The ND-fed mice were defined as the Control (Con group) and the HFD-fed mice were defined as the Obese mice with normoxia (Ob group). Obese mice were assigned to two groups at random and continued to be provided with HFD under normoxic and hypoxic conditions for 24 days.

Obese mice with normoxia (Ob group) were randomly subdivided into three subgroups (Ob-d6, Ob-d15, Ob-d24 groups) based on the duration of normoxic exposure (6, 15, 24 days). Obese mice with hypoxia (Ob-H group) were also randomly subdivided into three subgroups (Ob-H-d6, Ob-H-d15, Ob-H-d24 groups) based on the duration of hypoxic exposure (6, 15, 24 days).

#### 2.1.6 Organ weights

The absolute weights of the testes, epididymides, and epididymal fat tissues of the mice were recorded.

#### 2.1.7 Processing of blood and testicular tissue samples

The animals were anesthetized *via* intraperitoneal injection with phenobarbital (30 mg/kg). The mice were killed through rapid cervical vertebra dislocation after blood specimens were collected from the eyeball between 7 a.m. and 9 a.m., followed by centrifugation at 3500 rpm for 30 min to collect serum samples to measure hormone, lipid, fasting blood glucose (FBG), and fasting insulin (FINS) levels. Then, testes, epididymides, and epididymal fat tissues were isolated and stored in liquid nitrogen for subsequent experiments, which include analyses of testicular OS indicators, proteomic analysis, and PRM validation on the testis samples.

### 2.2 Experimental design

**Figure 1:**
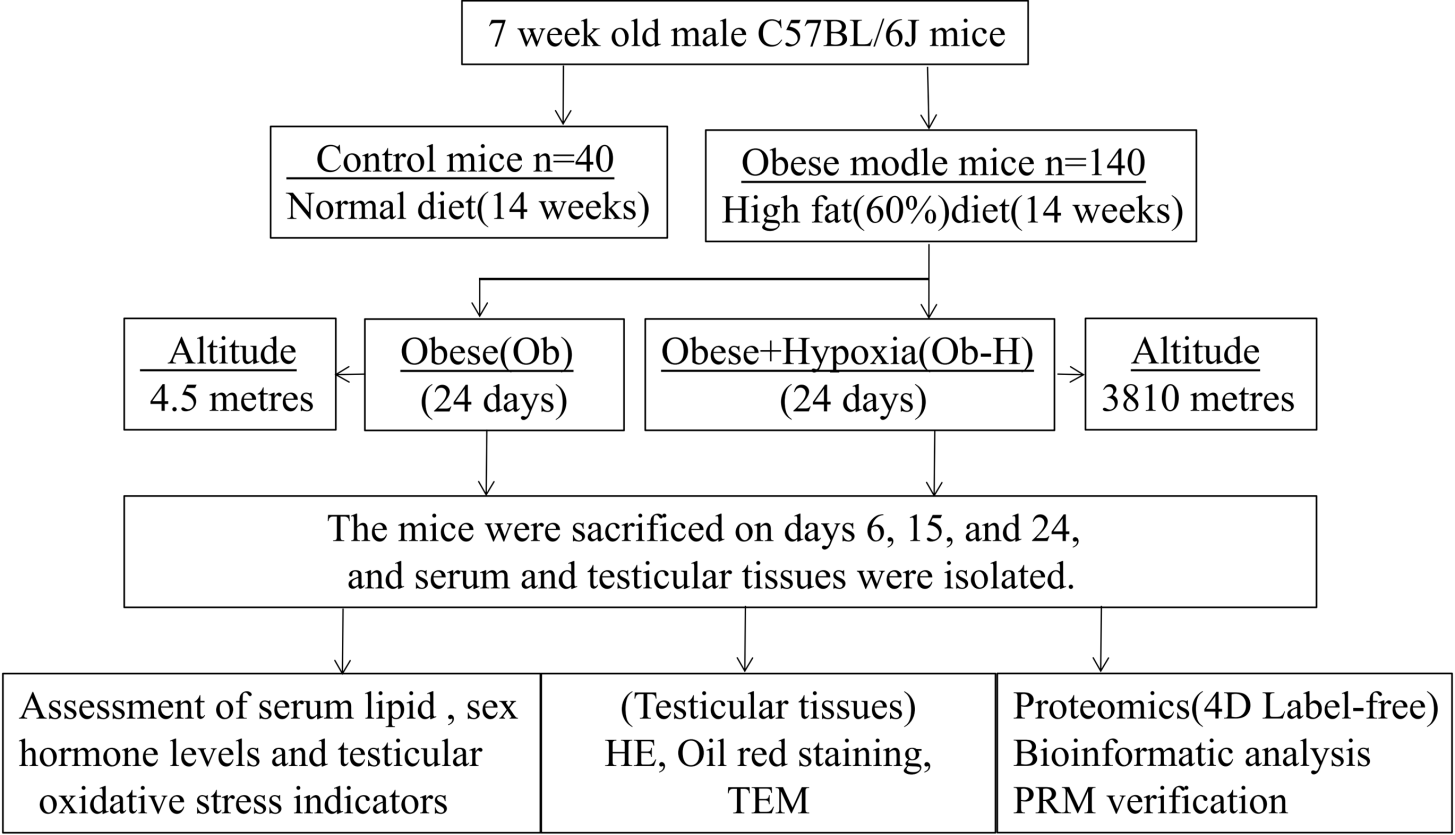
Flow chart of the experimental design.

The study design is shown in **Figure 1**.

### 2.3 Assessment of sex hormone levels, biochemical indexes, and hemoglobin level

Serum total testosterone (TT) and luteinizing hormone (LH) levels were assessed by using enzyme‐linked immunosorbent assay (ELISA) kits (Shanghai Enzyme-linked Biotechnology Co., Ltd, China) following the protocols outlined by the manufacturer. Isolated serum was tested for total cholesterol (TC), high-density lipoprotein cholesterol (HDL-C), and low-density lipoprotein cholesterol (LDL-C) *via* standard enzymatic colorimetric analysis. The level of serum triglycerides (TG) was measured using the colorimetric method with a Cobas C501 automated analyzer (Roche, Basel, Switzerland).

FBG level was checked using the hexokinase method. FINS level was assessed using mouse Insulin ELISA kit (SinoGene Biotech Co., Ltd, Beijing, China) based on the protocol recommended by the manufacturer. Insulin resistance was assessed through homeostasis model assessment of insulin resistance (HOMA-IR) using the formula: [FBG (mmol/L) × FINS (mU/L)]/22.5. Hemoglobin (HB) level was analyzed *via* electrochemical biosensing method with a hemoglobin meter (HemoCue Hb) using whole-blood specimens extracted from the tail tips of mice.

### 2.4 Analysis of testicular OS indicators

The testicular tissues were diluted in a 1:9 ratio of tissue weight (g) to normal saline volume (mL) before being homogenized in an ice water vessel. Centrifugation was performed on the homogenized solution in a high-speed refrigerated centrifuge at 2500 rpm for 10 min at 4°C. Then, homogenate supernatants were used for the detection of biomarkers of testicular OS, which include the activities of superoxide dismutase (SOD) and catalase (CAT) as well as malondialdehyde (MDA) and hydrogen peroxide (H_2_O_2_) content in the testicular tissues. The absorbance of OS indicators was measured at 532 nm (MDA), 550 nm (SOD), and 405 nm (CAT and H_2_O_2_) using the Huadong electronic DG5033A microplate reader (Huadong, Nanjing, China). The assay kits were purchased from the Jiancheng Bioengineering Institute (Nanjing, China).

### 2.5 Hematoxylin-eosin staining

The fresh testes were fixed in 10% paraformaldehyde (PFA) overnight for routine paraffin-embedded sections and stained with Hematoxylin-eosin staining (HE). Then, morphological changes in the testicular tissue sections on each slide were observed by scanning the entire field of view at 40× magnification. Finally, the areas of lesion in the testicular tissues selected randomly were photographed under 100× and 400× magnification using a digital trinocular microscopy system (Motic, BA210, Motic Corporation).

### 2.6 Oil red O staining

Frozen 5–6 μm-thick testicular tissue slices were processed for oil red O staining to evaluate lipid deposition in the testes. Each slide was initially observed at low power magnification (100×). Representative sections were photographed at 400× magnification. Representative sections of randomly chosen areas were photographed and analyzed. Meanwhile, testicular interstitial lipid quantification was performed using the image-Pro Plus 6.0 analysis system. The ratio of the red area to total area was used to quantify the formation of lipid deposition in the testes. All slices were observed using the BA210 Digital light microimaging system.

### 2.7 Transmission electron microscopy

Small pieces of testicular tissues were fixed in 2.5% glutaraldehyde, followed by fixation in 1% osmium tetroxide, dehydration in tandem acetone, and embedding in Epox 812 with extended infiltration time. Semithin sections were stained with methylene blue, and ultrathin slices were stained using uranyl acetate and lead citrate. Sections were observed and photographed using a JEM-1400-Flash Transmission electron microscopy (TEM).

### 2.8 Testicular tissue proteomic analysis

#### 2.8.1 Overview

Label-free quantitative proteomics is a protein quantification technique that does not rely on isotope labeling but instead uses the next-generation timsTOF Pro mass spectrometer (Bruker) to perform mass spectrometric analyses. Samples were quantitatively analyzed by using the trapped ion mobility spectrometry (TIMS) and parallel cumulative serial fragmentation (PASEF) scanning modes. On the basis of traditional 3D separation (retention time, mass to charge ratio, and ion strength), the collision cross-sectional area (CCS) of peptides was increased to achieve 4D separation detection. 4D-label free technique has a higher speed, sensitivity and stability. To minimize the individual differences between the mice, six isolated testicular samples from six mice in each group (*n* = 6) were mixed with three samples in each group for proteomic analysis.

#### 2.8.2 Sample preparation

The sample was transferred to 2 ml tubes with quartz sand and diluted with SDT buffer. Using the MP Fastprep-24 Automated Homogenizer, the lysate was homogenized twice at 6.0M/S for 30 seconds each time. A sonicator was used to homogenize the material, and then a 10-minute boil was performed. In addition to centrifuging at 14000g for 15 minutes, the supernatant was filtered through 0.22 μm filters. BCA Protein Assay Kit (P0012, Beyotime) was used to quantify the filtrate. Samples were stored at -80°C.

#### 2.8.3 Sodium dodecyl sulfate–polyacrylamide gel electrophoresis

Twenty micrograms of proteins was boiled for 5 min in loading buffer and then separated on 12.5% Sodium dodecyl sulfate–polyacrylamide gel electrophoresis (SDS-PAGE) gel and stained with Coomassie B for visualization.

#### 2.8.4 Filter-aided sample preparation

Each specimen was processed individually with 100 mM DTT at 100°C for 5 min using 50–200 µg of proteins. Ultrafiltrate processes were repeatedly performed to eliminate detergents, DTT, and residual low-molecular-weight components using UA buffer (8 M urea; 150 mM Tris HCl, pH 8.5). The samples were rinsed with UA buffer after 30-min incubation in the dark with 100 mM IAA. Washing was done thrice with the UA buffer and twice with NH_4_HCO_3_, and then the samples were washed again thrice with the UA buffer. Finally, the proteins were digested overnight at 37°C with trypsin in 50 mM NH_4_HCO_3_ buffer (40 μL), and then identified peptides were collected from the filter liquor.

#### 2.8.5 Mass spectrometry analysis

A nanoElute and timsTOF Pro equipped with a CaptiveSpray source (Bruker, Bremen, Germany) were used to analyze the samples. Peptide separation was carried out using an analytical column (25 cm × 75 μm) containing beads (1.6 μm C18) and an emitter tip (IonOpticks, Australia). Column temperature was maintained at 50°C using a columnar saggar (Sonation GmbH, Germany). We equilibrated the column using four column volumes, followed by a placement of samples into 100% buffer A (99.9% purified water, 0.1% FA) at 800 bar. The linear gradient separation (300 nL/min) was completed by holding buffer B for 1.5 h. The process was as follows: 2–22% buffer B for 75 min, 22–37% buffer B for 5 min, 37–80% buffer B for 5 min, and 80% buffer B for 5 min. The PASEF mode was applied to TimsTOF Pro. Range of mass (100–1700 m/z) was set, starting at 0.75 V.s/cm^2^ and ending at 1.4 V.s/cm^2^ (1/K0). Mass spectrometry (MS) parameters were as follows: Ramp time: 100 ms; Duty cycle (DC) varies: 100%; capillary voltage: 1500 V; dry gas: 3 l/min; dry temperature: 180°C. PASEF mode: MS/MS scans: 10 PASEF per cycle, total cycle time: 1.16 s, charge range (0–5); exclusive duration was 0.5 min; scheduling target intensity: 10000; intensity threshold: 2500 counts; CID collision energy: 20–59 eV.

#### 2.8.6 Data analysis

Max Quant software (version 1.6.17.0) and the UniProt database (Uniprot 17056_20210125) were used to analyze and search the MS data. Mass accuracy tolerance of ten parts per million (ppm) was selected as the mass window for a precursor search. The mass search was conducted according to an enzymatic cleavage rule of trypsin/P, maximum two missed cleavages sites, and a mass tolerance of 40 ppm for fragment ions. The false discovery rate (FDR) for peptide and protein was 1%. Standardized protein spectral intensities were used to determine protein abundance. Fold changes of more than 1.2 or less than 0.83 and *P*-values < 0.05 constituted the criteria used to define DEPs.

#### 2.8.7 Bioinformatics analysis

DEPs were classified according to the functional annotations and enrichment provided by the UniProt database (Swiss-Prot/TrEMBL, http://www.uniprot.org/) and the Gene Ontology (GO) database (http://www.geneontology.org/). GO enrichment analysis included GO terms subsumed under the biological process (BP), cellular component (CC), and molecular function (MF) categories. Pathway enrichment analysis of DEPs was performed using the Kyoto Encyclopedia of Genes and Genomes (KEGG) database (http://www.kegg.jp/). Fisher’s exact test was employed in the functional and pathway enrichment analysis of DEPs. In addition, the protein-protein interaction (PPI) network based on the DEPs was constructed using the STRING database (version 11.5; http://string-db.org/).

### 2.9 Parallel reaction monitoring validation of proteomics

Parallel reaction monitoring (PRM) is an expression abundance relative or absolute quantification technique for target protein or peptide based on high-resolution mass spectrometry. In the current study, ten DEPs of interest were selected according to the enriched KEGG pathways and networks to be validated independently by PRM. Quantitative analysis of proteins using PRM mainly includes protein extraction, protein quantification, protein gel electrophoresis, trypsin digestion, liquid chromatography–tandem mass spectrometry (LC-MS/MS) analysis, PRM parent ion scanning, and PRM data analysis. The MS parameters were as follows (i): Full-MS (scan range = 350–1800 m/z, resolution = 60,000, AGC target = 3e6, maximum injection time = 50 ms) (ii); PRM (resolution = 30,000, AGC target = 2e5, maximum injection time = 50 ms, loop count = 10, isolation window = 2.0 m/z, NCE = 27 eV).

### 2.10 Statistical analysis

All the data were analyzed using SPSS (version 20.0) and plotted with GraphPad Prism (version 6.0). One-way ANOVA or two-way ANOVA was employed for the statistical analysis of grouped data. Data were presented as mean ± standard error of the mean (SEM). The statistical significance level was set at α = 0.05.

## 3 Results

### 3.1 General conditions of mice with HFD (obese model)

**Figure 2:**
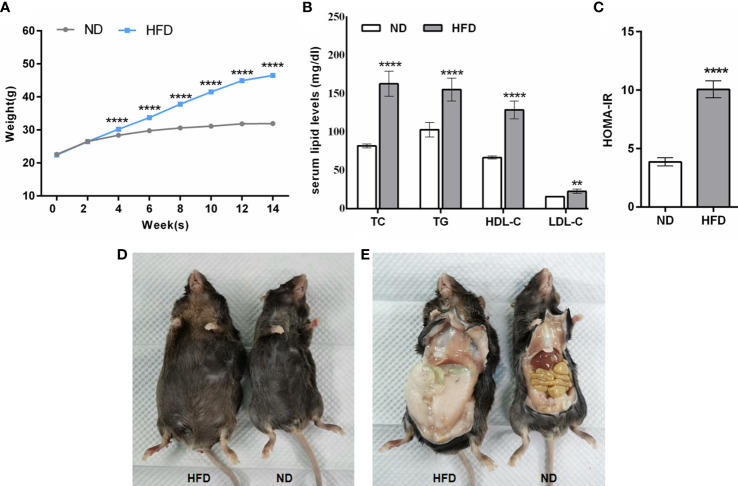
General conditions of mice fed with HFD. **(A–C)** The comparison of body weight, serum lipid levels, and HOMA-IR between mice fed with ND and HFD. **(D, E)** Comparison of body somatotype and adipose tissue between mice fed with ND and HFD. Data are represented as mean ± SEM. ND, *n* = 40; HFD, *n* = 140; **(B, C)**: *n* = 15; **P < 0.01 vs ND, ****P < 0.0001 vs ND. ND, normal diet; HFD, high-fat diet.

Animals fed with HFD gained significant body weights after the 4th week, and this trend continued with prolonged feeding times (*P <* 0.0001, **Figure 2A**) in contrast to animals fed with ND. At the end of the 14th week, the mean body weight of mice with HFD was 1.44-fold higher than that of the ND group and showed a stable trend (**Figure 2A**). Correspondingly, there was a significant increase in serum lipid (TC, TG, HDL-C, LDL-C) levels in the HFD group compared to the levels in ND group (*P* < 0.0001, **Figure 2B**). Similar observations were seen in the results of HOMA-IR (*P* < 0.0001, **Figure 2C**). In addition, apparent changes in body somatotype and adipose tissue were also directly observed between mice fed with ND and HFD (**Figures 2D, E**). Overall, these results suggested that the obese mouse model was successfully replicated.

### 3.2 General status of obese mice after chronic high-altitude hypoxia exposure

Some obese mice showed poor general states (reduced spontaneous activities, mild delays in response, and slightly fluffy fur) during the first 1 to 2 days of high-altitude exposure. After 2–3 days, the general condition of mice gradually improved in the mice as inferred from increased spontaneous activities, faster response times, and smooth and flat fur.

### 3.3 Impacts of chronic high-altitude hypoxia on HB, body weight, and organ weight in obese male mice

**Figure 3:**
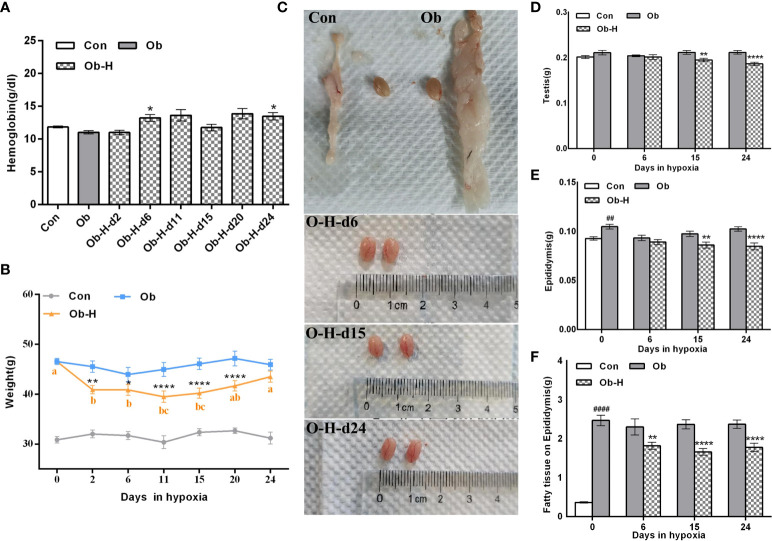
Impacts of chronic hypoxia exposure on HB, body weight, and organ weight in Ob mice. **(A)** Comparison of HB between the Ob and Ob-H groups. **(B)** Comparison of body weight between the Ob and Ob-H groups. **(C)** Comparison of testicular appearance between the Ob and Ob-H groups. **(D, E)** Comparison of the absolute weight of the testes and epididymides between the Ob and Ob-H groups. **(F)** Comparison of the absolute weight of the epididymal fat between the Ob and Ob-H groups. Data are represented as mean ± SEM. **(A)** *n* =10–19; **(B)** *n* = 6–19; **(D–F)** *n* = 16–22; ^##^ *P* < 0.01 vs Con, ^####^ *P* < 0.0001 vs Con; *P < 0.05 vs Ob, **P < 0.01 vs Ob, ****P < 0.0001 vs Ob. Labeling with the same alphabetic letter represents an absence of differences between groups. Con, Control group; Ob, Obese group; Ob-H, Obese-Hypoxia group.

There was an obvious increase in the HB level of obese mice after chronic high-altitude hypoxia exposure (Ob-H groups) compared to the HB level under a normoxic condition (Ob groups), especially on day 6 and day 24 of the high-altitude hypoxia exposure (*P* < 0.05, **Figure 3A**). As for body weight, compared with the corresponding Ob groups, there was rapid weight loss when obese mice were exposed to a hypoxic environment for the first two days (*P* < 0.05, **Figure 3B**). However, after that, the rate of weight loss slowed down significantly. We further observed that the body weight of the obese mice in the hypoxia groups was stable during days 2 to 20 of hypoxia exposure (*P* > 0.05, **Figure 3B**). In addition, the body weight of obese mice showed an increasing trend starting from 11 days of hypoxia and reached comparable levels to that of normoxic obese mice at 24 days of hypoxia. Meanwhile, we found that the body weight of the mice across all Obese-Hypoxia groups was still noticeably 1.2-fold greater than that in Control groups (*P* < 0.05, **Figure 3B**). These results implied that the obese mice could still maintain their obesity status despite a certain degree of weight loss under chronic hypoxia. In addition, the testes in Ob-H groups exhibited hyperemia compared with those in the corresponding Ob groups (**Figure 3C**). The absolute weight of testis and epididymis was lower in the Ob-H groups (on day 15 and 24 of hypoxic exposure) than that of testis and epididymis in the corresponding Ob groups (*P* < 0.05, **Figures 3D, E**). The absolute weight of epididymal fat in mice in all Obese-Hypoxia groups was lower than that in mice in the corresponding Obese groups (*P*<0.05, **Figure 3F**). However, the absolute weight of epididymal fat across all Obese-Hypoxia groups was still noticeably greater than that of the Control group (*P*<0.05, **Figure 3F**). The changes in visceral fat in the epididymis also suggested the persistence of visceral obesity in obese mice after chronic high-altitude hypoxia exposure.

### 3.4 Impacts of chronic high-altitude hypoxia on testicular histology of obese male mice

**Figure 4:**
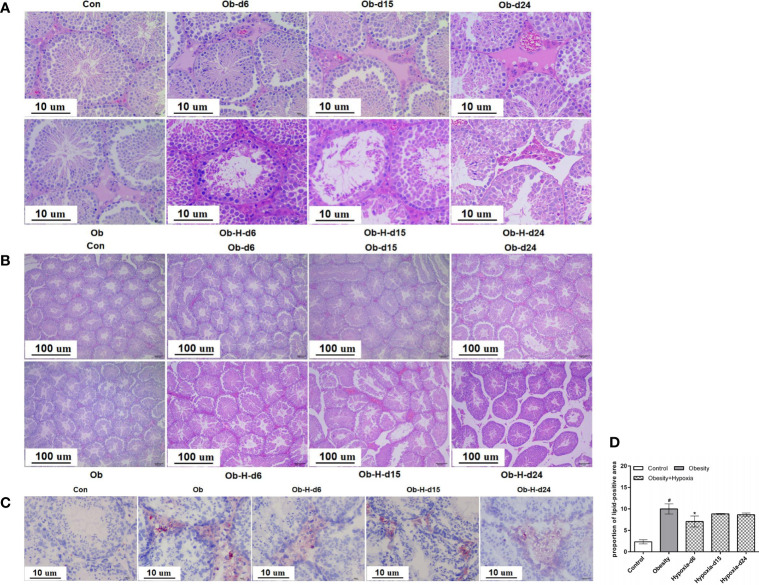
Impacts of chronic high-altitude hypoxia on the testicular histology of obese male mice. **(A, B)** The representative light microscopic histology images of testicular tissues. Magnification: 100×, scale bar = 100 μm; 400×, scale bar = 10 μm. **(C)** Lipid accumulation in the interstitial tissues of testes by oil red O staining. Magnification: 400×, scale bar = 10 μm. **(D)** Quantitative analysis of the lipid-positive areas in testicular interstitial tissues; **(A, B)** *n* = 5; **(C, D)** *n* = 4- 6. Data are represented as mean ± SEM. ^#^ *P* < 0.05 vs Con; *P < 0.05 vs Ob. Con, Control group; Ob, Obese group; Ob-H, Obese-Hypoxia group.

HE staining suggested that the interstitial space of the testicular sections slightly increased and the arrangement of LCs was disordered in the Ob mice in contrast to that in the Con mice (**Figures 4A, B**). Notably, the condition of increased interstitial space and disordered arrangement of LCs in Ob-H mice was further exacerbated relative to the Ob group mice. Meanwhile, the blood vessels of the interstitial tissues in the testis appeared partly dilated and congested in the Ob-H mice. In addition, disorganized and atrophied seminiferous tubules and even necrotic spermatogenic tubules were observed in the Ob-H mouse tissue samples (**Figures 4A, B**). These results implied that high-altitude hypoxia exposure aggravated testicular histopathological damage in obese male mice.

Oil red O staining revealed that lipid accumulation mainly occurred in the interstitial tissues of testes (**Figure 4C**). Further quantitative analysis found that the Ob mice exhibited greater lipid accumulation in the testicular interstitial tissues than the Con mice did (*P* < 0.05, **Figure 4D**). However, the proportion of lipid-positive areas was slightly lower in the Ob-H-d6 mice than in the Ob mice, and no further differences were observed in the Ob-H-d15 and Ob-H-d24 mice relative to the mice in the corresponding Ob subgroups (**Figure 4D**). These results confirmed that lipid deposition still persisted in the testicular interstitial space of obese male mice even in chronic high-altitude hypoxia.

### 3.5 Chronic high-altitude hypoxia decreased TT levels in obese male mice

**Figure 5:**
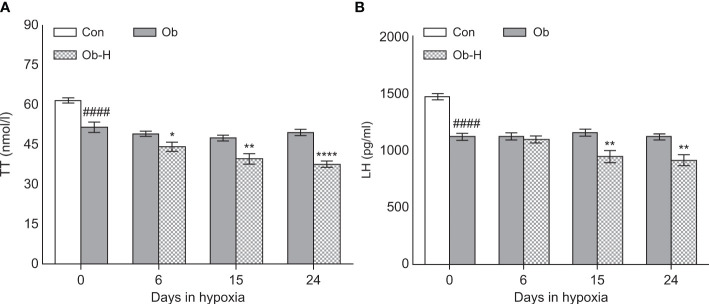
Chronic high-altitude hypoxia exposure decreased TT levels in obese male mice. **(A)** serum TT concentration, **(B)** serum LH concentration. Data are represented as mean ± SEM. **(A, B)**:*n* = 12–22, ^####^ *P* < 0.0001 vs Con; *P < 0.05 vs Ob, **P < 0.01 vs Ob, ****P < 0.0001 vs Ob. Con, Control group; Ob, Obese group; Ob-H, Obese-Hypoxia group.

Serum TT level is the main diagnostic method for male hypogonadism. To analyze the effects of chronic hypoxia on the hormone concentration, obese mice were exposed to high-altitude hypoxia environment for 24 days. Both serum TT and LH levels were lower in the Ob-H group mice on day 15 and 24 than in the mice in the corresponding Ob groups (*P* < 0.05, **Figures 5A, B**). However, the decrease in serum TT level was not accompanied by a corresponding decrease in LH level in Ob-H-d6 group unlike in the corresponding Ob-d6 group (*P* < 0.05, **Figures 5A, B**). These results indicated that male hypogonadism may either be primary, secondary, or both in this context.

### 3.6 Chronic high-altitude hypoxia aggravated testicular tissue OS in obese male mice

**Figure 6:**
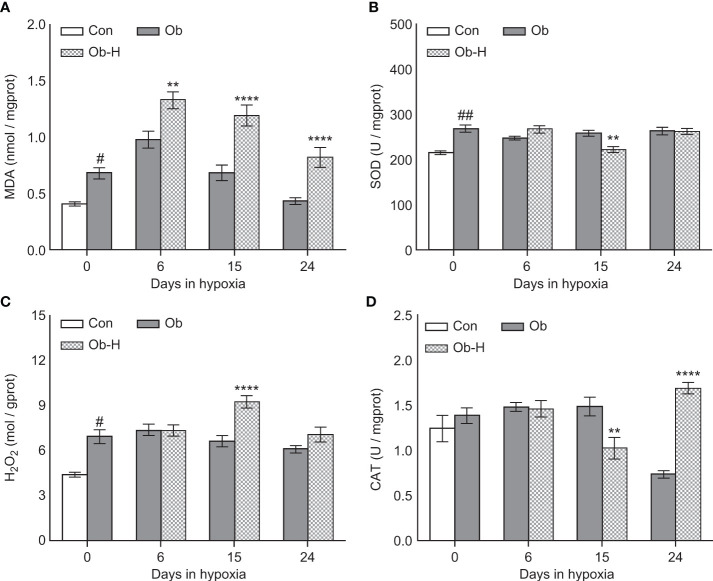
Chronic high-altitude hypoxia exposure aggravated testicular tissue oxidative stress in obese male mice. **(A)** MDA; **(B)** SOD; **(C)** H_2_O_2_; **(D)** CAT. Data are represented as mean ± SEM. **(A–D)** *n* = 12–22, ^#^ *P* < 0.05 vs Con, ^##^ *P* < 0.01 vs Con, **P < 0.01 vs Ob, ****P < 0.0001 vs Ob. Con, Control group; Ob, Obese group; Ob-H, Obese-Hypoxia group.

We determined MDA, SOD, H_2_O_2_, and CAT levels in testicular tissues to explore the impacts of chronic high-altitude hypoxia on testicular tissue OS in obese male mice. The MDA level in the testis was higher in the Ob group than in the Con group (*P* < 0.05, **Figure 6A**). Notably, the MDA level in the testis was appreciably higher in all three Ob-H subgroups than in the corresponding Ob subgroups (*P* < 0.05, **Figure 6A**). Additionally, SOD activities in the Ob-H-d15 mice showed a reduction compared to those in the Ob-d15 mice (*P* < 0.05, **Figure 6B**). The testicular H_2_O_2_ content exhibited a slight rise in the mice from the Ob group compared to that in Con mice (*P <* 0.05, **Figure 6C**) and a significant increase in the Ob-H-d15 mice compared to that in the Ob-d15 mice (*P* < 0.05, **Figure 6C**). Meanwhile, CAT activities in the Ob-H-d15 mice were lower than those in the Ob-d15 mice (*P <* 0.05, **Figure 6D**). Nonetheless, the CAT level in Ob-H-d24 mice showed an increase when compared to the CAT level in Ob-d24 mice (*P <* 0.05, **Figure 6D**). Altogether, these results provided a direct evidence that chronic high-altitude hypoxia exposure aggravated testicular tissue OS in obese male mice, which is highlighted by the fact that testicular MDA levels were significantly increased in mice in all Ob-H groups compared to those in the corresponding Ob groups.

### 3.7 Impacts of chronic high-altitude hypoxia on the mitochondria of LCs in obese male mice

**Figure 7:**
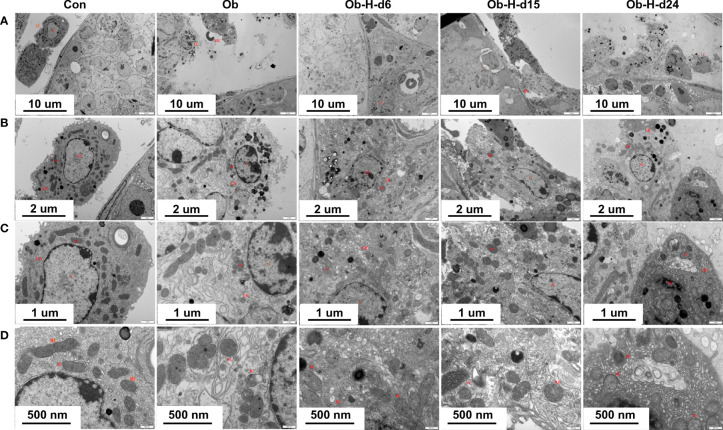
Impacts of chronic high-altitude hypoxia on the mitochondria of LCs in obese male mice. Representative TEM images showing the morphology of mitochondria, nucleus, and membrane from each group. Magnification: **(A)** 3,000×, scale bar = 10 μm; **(B)** 8,000×, scale bar = 2 μm; **(C)** 15,000×, scale bar = 1 μm; **(D)** 30,000×, scale bar = 500 nm. (A, B ,C ,D): *n* = 3; Mitochondrial (Mi); Nucleus (N); Endoplasmic reticulum (ER); Red blood cell (RBC); Leydig cells (LCs). Con, Control group; Ob, Obese group; Ob-H, Obese-Hypoxia group.

Mitochondrion is the essential organelle for ATP generation and testosterone production, and it also plays a major role in cellular ROS production. Therefore, mitochondrial and ultrastructural changes were observed in this study using TEM analysis (**Figures 7A–D**), which showed the presence of mitochondria with irregular nuclei, discontinuous cell membrane, and mild swelling in some areas of LCs in Ob mice compared to the mitochondria of the Con mice. Remarkably, our study demonstrated that the mitochondria with irregular nuclei, discontinuous cell membrane, and mild swelling in LCs gradually increased in severity with hypoxic exposure relative to those in the LCs of the Ob mice. Meanwhile, the disruption of mitochondrial crests, cytoplasmic vacuolization, and LC necrosis were also observed locally in samples from the Ob-H groups. These results showed that chronic high-altitude hypoxia exposure exacerbated mitochondrial damage in LCs of obese mice.

### 3.8 Proteomic profiling through 4D label-free analysis

#### 3.8.1 Overview

Decreases in serum testosterone are generally caused by extrinsic factors, intrinsic factors, or both. The HPG axis is required for the control of external factors. Intrinsic factors of testosterone production rely on substrate (cholesterol) availability, trafficking, and steroidogenic enzyme activities (16). Our study showed that the reduction in testosterone production was independent of the LH levels on day 6 of high-altitude hypoxia exposure in obese male mice. This indicates a more local and intrinsic testicular effect. Therefore, taking into account testicular pathological changes, testes from the two comparison groups (Ob/Con; Ob-H-d6/Ob) were ultimately chosen for proteomic analysis to explore in depth the intrinsic testicular mechanisms underlying the perturbation of testosterone synthesis in obese male mice under chronic high-altitude hypoxia exposure.

#### 3.8.2 Qualitative and quantitative analyses of proteins in testicular tissues

**Figure 8:**
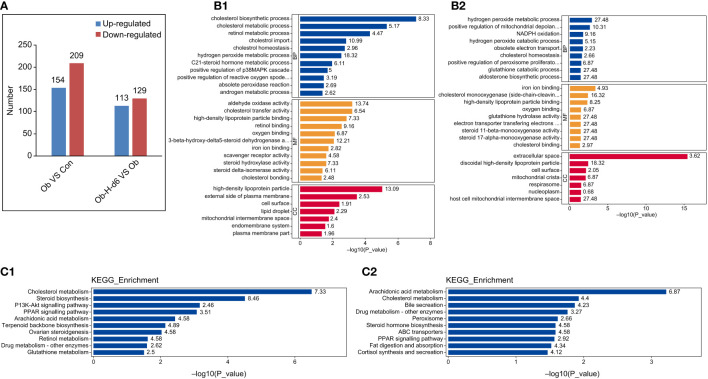
Functional and pathway enrichment analysis of DEPs. **(A)** The distribution of the number of DEPs in histogram (Ob/Con and Ob-H-d6/Ob). Blue and red indicate significantly upregulated and downregulated DEPs, respectively. **(B1, 2)** GO functional analysis of DEPs in the Ob/Con and Ob-H-d6/Ob comparison groups. Blue, yellow, and red represent biological process (BP), molecular function (MF), and cellular component (CC), respectively. **(C1, 2)** KEGG pathway analysis of DEPs (Ob/Con and Ob-H-d6/Ob). Con, Control group; Ob, Obese group; Ob-H, Obese-Hypoxia group.

MS analysis revealed a total of 1,526,843 spectrum proteins, 861,489 matched spectrum proteins, 74,770 peptides, 72,553 unique peptides, 6651 identified proteins, and 6031 quantifiable proteins. In our study, there were 363 DEPs in the Ob/Con comparison group, which contained 154 upregulated and 209 downregulated proteins. There were 242 DEPs in the Ob-H-d6/Ob comparison group, which contained 113 upregulated and 129 downregulated proteins. (**Figure 8A**).

#### 3.8.3 Functional and pathway enrichment analysis of DEPs

Biological functional enrichment analyses of the DEPs were performed using GO and KEGG database to investigate potential mechanisms influencing testosterone production in obese mice under chronic high-altitude hypoxia exposure.

GO functional analysis revealed that biological processes likely of relevance to testosterone production were altered in the Ob/Con and Ob-H-d6/Ob comparison groups (*P* < 0.05, **Figures 8B1, 2).** These include the cholesterol biosynthetic process, cholesterol homeostasis, and steroid hormone metabolic process. Meanwhile, positive regulation of ROS metabolic process was enriched in the Ob/Con comparison group. Response to oxidative stress and positive regulation of mitochondrial depolarization were enriched in the Ob-H-d6/Ob comparison group; this was also the case with molecular functions involving cholesterol binding, cholesterol monooxygenase (side-chain-cleaving) activity, and iron ion binding. In terms of cellular component, DEPs related to the above BP and MF categories were distributed in the cytoplasm, organelles (mitochondria, endoplasmic reticulum, and lipid droplets), plasma membrane, and the nucleus.

The KEGG pathway enrichment analysis found that the pathways associated with steroid biosynthesis such as cholesterol biosynthesis, cholesterol transport, steroid hormone biosynthesis processes, PPAR signaling pathway, arachidonic acid metabolism, glutathione metabolism as well as retinol metabolic pathway were enriched in the Ob/Con and Ob-H-d6/Ob comparison groups (*P* < 0.05, **Figures 8C1, 2**).

### 3.9 Validation of proteomic results

#### 3.9.1 Selection of candidate DEPs for validation

**Figure 9:**
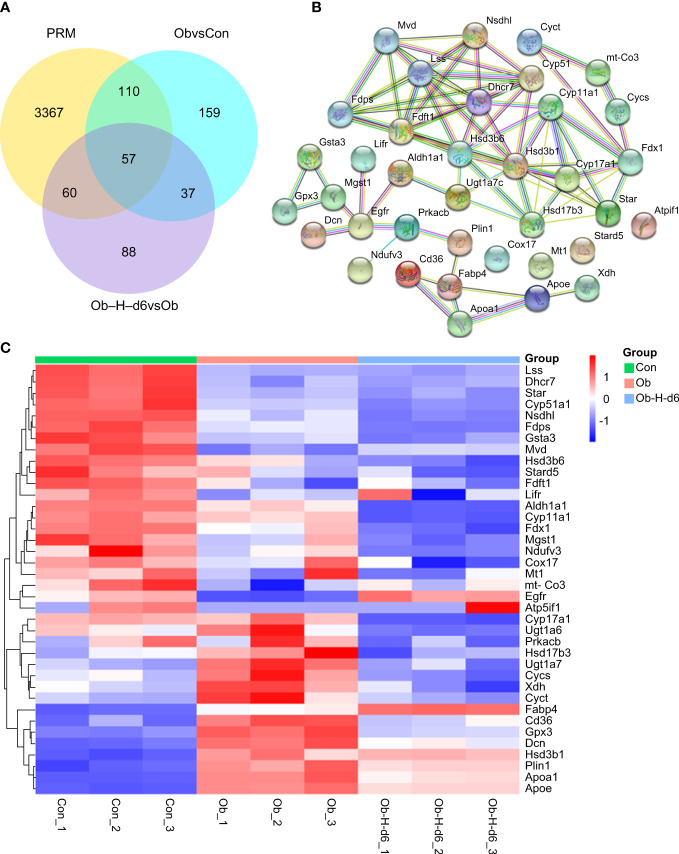
**(A)** Number of DEPs that could be accurately quantitated by PRM in the Ob/Con and Ob-H-d6/Ob comparison groups. **(B)** PPI network analysis using the STRING database for 38 DEPs likely related to testosterone production. **(C)** Heatmap analysis for the 38 representative DEPs. Con, Control group; Ob, Obese group; Ob-H, Obese-Hypoxia group.

DEPs for validation were selected based on the (i) results of the bioinformatics analysis; (ii) biological relevance and novelty to testosterone production; and (iii) DEPs that can be accurately quantified by PRM (**Figure 9A**). Thus, 38 DEPs likely related to testosterone production were selected for PPI network analysis using the STRING database. Network analysis results indicated more than expected interconnection in the function of DEPs (*P* < 1.0e-16) (**Figure 9B**). A heatmap analysis for these 38 DEPs was subsequently performed (**Figure 9C**).

#### 3.9.2 Representative DEPs for PRM verification

**Figure 10:**
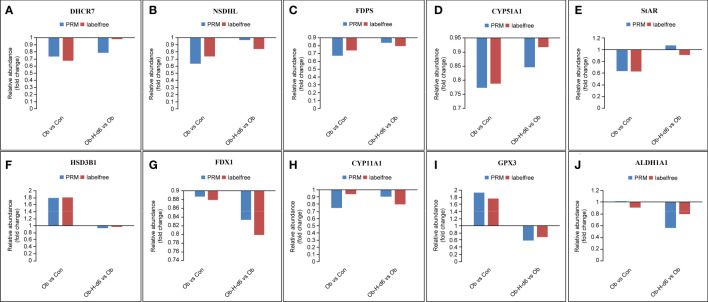
Expression patterns of ten representative DEPs using 4D label-free analysis and PRM. **(A)** DHCR7, **(B)** NSDHL, **(C)** FDPS, **(D)** CYP51A1, **(E)** StAR, **(F)** HSD3B1, **(G)** FDX1, **(H)** CYP11A1, **(I)** GPX3, **(J)** ALDH1A1. Con, Control group; Ob, Obese group; Ob-H, Obese-Hypoxia group.

Based on the above analysis, ten representative DEPs, namely, 7-dehydrocholesterol reductase (DHCR7), NAD(P) dependent steroid dehydrogenase-like (NSDHL), farnesyl diphosphate synthetase (FDPS), lanosterol 14-alpha demethylase (CYP51A1), StAR, HSD3B1, FDX1, CYP11A1, glutathione peroxidase-3 (GPX3), and aldehyde dehydrogenase family-1 (ALDH1A1) were eventually chosen for PRM validation (*P* < 0.05, **Figure 10**). The PRM validation outcomes indicated that the expression of protein DHCR7, NSDHL, CYP51A1, and StAR was reduced and the expression of protein HSD3B1 and GPX3 was upregulated in the Ob group relative to those in the Con group. However, the expression of FDX1, CYP11A1, GPX3, and ALDH1A1 in the Ob-H-d6 group was reduced compared with the expression of the same proteins in the Ob subgroup. It is noteworthy that the expression of protein FDPS in the Ob group was reduced relative to its expression in the Con group and a further decrease of FDPS was seen in the Ob-H-d6 group relative to its expression in the Ob subgroup. Combined with protein feature analysis, these ten proteins were revealed to be involved in cholesterol biosynthesis, cholesterol transport, steroid hormone biosynthesis processes, response to oxidative stress, and retinol metabolism pathways or biological processes.

The PRM validation results of most of the ten representative DEPs related to testosterone production were consistent with the results of the MS analysis, thereby demonstrating the veracity and dependability of the proteomic data. Nevertheless, inherent differences in the distinct inspection technologies may explain the discrepancies between 4D label-free proteomic and PRM approaches, just as there are inherent technical differences between tandem mass tag and PRM approaches (33).

## 4 Discussion

This work explored how chronic high-altitude hypoxia exposure influenced testosterone production in obese male mice. Our findings demonstrated that chronic high-altitude hypoxia exposure induced a further lowering of testosterone level, an increase in testicular OS, and histological damages compared to obese mice under hypoxia conditions. Testicular lipotoxicity was sustained in obese mice despite a certain degree of weight loss under chronic hypoxia conditions. Chronic high-altitude hypoxia exposure was likely to exacerbate the risk of low testosterone level in obese mice by altering expressions of key proteins involved in steroid hormone biosynthesis, cholesterol biosynthesis, and OS responses.

Firstly, to study the influence and mechanism of hypoxia exposure on testosterone production in obese male mice, the HFD-induced obese mouse model was successfully replicated. As expected, the body weight of mice in the HFD group increased significantly, concomitant with hyperlipemia and testicular lipid deposition. Notably, rapid weight loss accompanied by poor general status was observed in obese male mice for the first two days of hypoxia exposure. After that, the rate of weight loss slowed down significantly, and general status improved gradually. The change reflected a complex process that the obese mice acclimated gradually to acute high-altitude hypoxia. Meanwhile, we found the body weight of obese mice remained noticeably higher than that of control mice by more than 1.2 times within 24 days of hypoxia despite a body weight loss compared with the normoxia obese mice. In addition, the amount of adipose tissue is an important indicator of obesity, and gonadal visceral fat is an important fat pool in rodents. Therefore, changes in visceral fat on the epididymis also suggested the persistence of visceral obesity in obese mice after exposure to chronic hypoxic environments. These results showed that the obese mice could still maintain their obesity status despite a certain degree of body and epididymis fat weights losses after chronic high-altitude exposure.

The intrinsic abundance of highly unsaturated fatty acids as well as active metabolism and mitogenesis in testicular tissues render them vulnerable to OS-induced pathologies (34). Moreover, testicular interstitial O2 tension is approximately 20% of testicular arterial oxygen pressure (i.e., 12 to 15 mm Hg) in rats (35). Therefore, the low testicular interstitial O2 tension is another critical factor contributing to the susceptibility of testicular tissue to oxidative stress. Long-term high-fat diet can damage the antioxidant defense system and mitochondrial metabolism (36, 37), thereby causing oxidative damage to testicular LCs (38) and lowering testosterone level. Similarly, we demonstrated that HFD induced OS damage in testicular tissues accompanied by a decrease in testosterone levels. It has recently been shown that acute hypoxic exposure usually leads to an increase in adaptive testosterone synthesis (39). Excessive ROS accumulates in testicular cells in prolonged hypoxic environment, resulting in LCs damage, thereby causing low testosterone output (27). Our findings showed the obese mice could still maintain their obesity status under chronic hypoxia. However, the MDA level in the testis was appreciably higher in all Obese-Hypoxia groups than that in the corresponding Obese groups, which indicated that chronic hypoxia was likely to promote testicular OS in obese mice. Meanwhile, our findings indicated that further lowering of testosterone levels occurred, accompanied by further testicular histopathology and mitochondrial damage of LCs in obese mice after chronic hypoxia exposure. The progression of testicular OS in obese mice exposed to hypoxia was likely related to electron leaks (40) in the mitochondrial electron transport chain (ETC) and the disturbance of cellular defense systems under hypoxia (41), thus ultimately promoting lipid peroxidation. In conclusion, these results suggested that the reduction in testosterone output in obese mice exposed to chronic hypoxia was related to a more drastic testicular OS in hypoxia may become a contributing factor to the decrease in testosterone levels. Furthermore, we found that the low testosterone levels were independent of the LH levels on day 6 of hypoxia, which suggested that the further decrease in testosterone production in obese mice exposed to hypoxia on day 6 is more dependent on intrinsic testicular factors than external factors. In fact, it is challenging to distinguish entirely ”primary” or “secondary” for male hypogonadism secondary to complex factors. Hypogonadism in obese male mice exposed to hypoxia might be primary, secondary, or both, which might depend on the extent of obesity and the pattern and duration of hypoxia exposure.

Two comparison groups (Ob/Con and Ob-H-d6/Ob) were chosen for proteomic analysis to investigate the intrinsic molecular mechanisms in the testis by which chronic hypoxia aggravates hypotestosteronemia in obese mice. Ultimately, ten representative DEPs were selected for PRM verification based on the results of the bioinformatics analysis, taking into consideration their biological relevance and novelty to testosterone production. These ten DEPs were mainly involved in shared critical pathways or biological processes in the two comparison groups, namely, steroid hormone biosynthesis, cholesterol biosynthesis, oxidative stress responses, and retinol metabolism.

### 4.1 Steroid hormone biosynthesis

Previous studies have reported that OS could disrupt mitochondrial homeostasis and could subsequently interfere with StAR protein transcription, which ultimately reduced steroid production (42). Our research also verified that HFD considerably downregulated the StAR protein expression, which was accompanied by testicular OS damage and the swelling of mitochondria in LCs relative to control. This implies that testicular OS induced by HFD may directly affect mitochondrial homeostasis in LCs thereby downregulating the expression of StAR protein, resulting in the decrease of testosterone synthesis. It is worth noting that the decrease in HSD3B1 expression in obese male mice was previously reported by Frank Cloutier et al. (43). However, our study found that the expression of HSD3B1 in the testis was upregulated in HFD-induced obese male mice and this observation was consistent with previous results in the literature (44). The reason for these contradictory findings remains obscure.

Hypoxic rats have been reported to display lower serum testosterone level and a downregulation of StAR and HSD3B1 in the testis (31). These changes were associated with an increase in oxidants and the upregulation of endoplasmic reticulum (ER) stress (30). Although our study found that chronic hypoxia exposure concomitantly exacerbated testicular OS damage and the decrease in testosterone level in obese male mice, we did not observe the downregulation of StAR and HSD3B1 in the testis at this point. Possible explanations include differences in both research objects and patterns of hypoxia exposure. Glutathione peroxidase (GPX) protects cells from the damaging effects of OS *via* the catalytic reduction of hydrogen peroxide and lipid peroxides (45). Our study found that the protein expression of the antioxidant GPX3 in the testis was significantly downregulated in obese male mice after chronic hypoxia exposure, which was likely to promote testicular OS indirectly. Remarkably, the protein expression of FDX1 and CYP11A1 in the testicular tissues of obese mice showed a synchronous downregulation after chronic hypoxia exposure. FDX1 acts as a redox partner and transfers electrons from NADPH to CYP11A1. This promotes the transformation of cholesterol to pregnenolone as the rate-limiting step in the process of steroid hormone biosynthesis (46). Excessive ROS levels affect steroid production by inhibiting CYP11A1 activity and reducing the levels of FDX1 as a cofactor for NADPH (11, 47). Therefore, the downregulation of testicular FDX1 and CYP11A1 expression in obese mice after hypoxia exposure strongly indicated that testosterone production was impaired at this point. Mitochondrial electron leak is one of the main sources of intracellular ROS production (48), and the reduction in oxygen levels under hypoxia can lead to electron leakage from the electron transport chain, which increases ROS production (49, 50). Therefore, we speculated that chronic hypoxia exposure induced the accumulation of ROS, which could interfere with the function of FDX1 and CYP11A1 steroid synthase, and thus exacerbate hypotestosteronemia in obese mice.

### 4.2 Cholesterol biosynthesis

Cholesterol is the main substrate for testosterone biosynthesis and is derived from the cholesterol in blood circulation and from *de novo* synthesis (17, 51). The testes primarily exploit *de novo* cholesterol as the substrate for steroid biosynthesis (52, 53). *In vitro* and ex vivo studies indicated that exhausted cholesterol in LCs accounted for the decrease in testosterone production (54). It has been reported that the mRNA expression of *Cyp51* (55) and the expression of HMGCR (56), which are involved in *de novo* cholesterol synthesis, were downregulated in the liver after a short‐term HFD. Our results discovered that the protein expression of *Dhcr7*, *Fdps*, *Nsdhl*, and *Cyp51a1* genes (57), which code for the vital enzymes in *de novo* cholesterol biosynthesis, were downregulated in the testis after HFD, which might partly explain the observed low testosterone production following HFD as deficiency in *de novo* cholesterol directly impacts testosterone synthesis.

Interestingly, previous studies have reported that OS induced by low oxygen level affects gonadal hormone production through cholesterol suppression and other pathways, causing gonad dysfunction (58). In the present study, the testicular FDPS protein expression in obese mice was further downregulated after chronic hypoxia exposure. Upon noting that FDPS is a key enzyme in *de novo* cholesterol biosynthesis (59) that catalyzes the formation of farnesyl diphosphate (FPP), we reasoned that the downregulation of FDPS in the testis may give rise to diminished levels of intracellular cholesterol, partially explaining the further lowering of testosterone level in obese mice after chronic hypoxia exposure due to the lack of cholesterol substrate for testosterone synthesis. However, whether OS in the testes of obese mice exposed to hypoxia is involved in the impairment of *de novo* cholesterol biosynthesis remains to be elucidated.

### 4.3 Retinol metabolism

ALDH1A1 is a crucial rate-limiting enzyme that converts retinaldehyde into retinoic acids (RA) (60). *In vitro* studies have shown that RA and retinol can enhance the expression of StAR protein and CYP17A1, thereby regulating testosterone synthesis (61, 62). The expression of antioxidant genes, including Aldh1a1, has been reported to be upregulated by the antioxidant transcription factor nuclear factor erythroid 2-related factor 2 (NRF2) in renal tissues after ischemia reperfusion to promote ischemic preconditioning (63), stressing at the antioxidative nature of ALDH1A1 under tissue hypoxia. Our study found that long-term HFD did not induce the compensatory elevation of testicular ALDH1A1 in obese mice; however, the expression of ALDH1A1 was downregulated in the obese mice after chronic hypoxia exposure. We speculated that the reduced expression of ALDH1A1 in the testis may promote testicular OS. In addition, the decreased RA production caused by the low ALDH1A1 expression in the testis may downregulate testicular steroid synthase expression (61). This might also partially explain the further reduction of testosterone synthesis in obese male mice after chronic hypoxia exposure.

## 5 Conclusion

Chronic high-altitude hypoxia exposure exacerbated testicular OS and lowered testosterone production in obese mice, accompanied by aggravated testicular histopathology and mitochondrial damage of LCs. This study is the first to use quantitative proteomics to investigate the effect of chronic hypoxia on the testicular tissue proteome of obese mice. Several significant functional terms and pathways related to testosterone production were altered in Obese/Control and Obese-Hypoxia/Obese groups. The downregulation of StAR, DHCR7, NSDHL, CYP51A1, and FDPS protein in the testes of obese male mice could partially explain the low testosterone production that was observed. Further, the downregulation of FDX1, CYP11A1, FDPS, ALDH1A1, and GPX3 protein occurred within shared pathways or biological processes, potentially accounting for the further lowering of testosterone production in obese male mice after chronic hypoxia exposure. This study provides novel insights into the protein dysregulation mechanism behind testosterone production in obese male mice after chronic high-altitude hypoxia exposure. However, the present study has its limitations. For instance, the in-depth mechanism of the DEPs changes in obese male mice with hypoxia was not able to be clarified. Further research is encouraged to determine such molecular mechanism in order to present a rationale for the development of potential targeted therapeutics for the dysfunction of testosterone production in this high-risk group.

## Data availability statement

The mass spectrometry proteomics data have been deposited to the ProteomeXchange Consortium (http://proteomecentral.proteomexchange.org) via the iProX partner repository with accession number PXD037055.

## Ethics statement

The animal study was reviewed and approved by Animal Ethics Committee of Qinghai Provincial People’s Hospital affiliated to Qinghai University (No. 2021-39). All experimental procedures were performed in strict accordance with the National Institutes of Health Guide for the Care and Use of Laboratory Animals.

## Author contributions

Conceptualization: SW and FL. Methodology: SW, YW, and CH. Validation: SW, YW, and CH. Formal analysis: YW and CH. Investigation: SW and CH. resources: SW and FL. data curation: YW and CH. writing—original draft preparation: SW. writing—review and editing: FL. Visualization: SW. supervision: FL. project administration: FL. All authors have read and agreed to the published version of the manuscript. All authors contributed to the article and approved the submitted version.

## Funding

This research was funded by Qinghai Provincial Science and Technology Department, grant number 2022-ZJ-776. The funder was not involved in the study design, collection, analysis, interpretation of data, the writing of this article, or the decision to submit it for publication.

## Acknowledgments

We thank Shanghai Genechem Co., Ltd for supporting the proteomic analysis. Shanghai Genechem Co., Ltd. did not fund this study but provided technical services on proteomic analysis.

## Conflict of interest

The authors declare that the research was conducted in the absence of any commercial or financial relationships that could be construed as a potential conflict of interest.

## Publisher’s note

All claims expressed in this article are solely those of the authors and do not necessarily represent those of their affiliated organizations, or those of the publisher, the editors and the reviewers. Any product that may be evaluated in this article, or claim that may be made by its manufacturer, is not guaranteed or endorsed by the publisher.
