# Corrigendum: Proteomic analysis reveals proteins and pathways associated with declined testosterone production in male obese mice after chronic high-altitude exposure

**DOI:** 10.3389/fendo.2023.1250527

**Published:** 2023-07-17

**Authors:** Shuqiong Wang, Youwen Wei, Caiyan Hu, Fang Liu

**Affiliations:** ^1^Research Center for High Altitude Medicine, Qinghai University, Xining, China; ^2^Key Laboratory of High Altitude Medicine, Ministry of Education, Xining, China; ^3^Key Laboratory of Application and Foundation for High Altitude Medicine Research in Qinghai Province, Qinghai-Utah Joint Research Key Lab for High Altitude Medicine, Xining, China; ^4^Department of Endocrinology, Qinghai Provincial People’s Hospital, Xining, China; ^5^Department of Plague Prevention and Control, Qinghai Institute for Endemic Disease Prevention and Control, Xining, China; ^6^Department of Laboratory Medicine, Baoding First Central Hospital, Baoding, China; ^7^Department of Biochemistry, Medical College, Qinghai University, Xining, China

**Keywords:** testosterone, obese, hypoxia, testis, proteomics, oxidative stress

In the published article, there were several unit errors and omissions of letter information during the text editing process in the **Materials and methods** section. The corrections to this section are as follows:

A correction has been made to **Materials and methods**, *2.8.2 Sample preparation*, Line 7. This sentence previously stated, “the supernatant was filtered through 0.22 m filters”. The corrected sentence is “the supernatant was filtered through 0.22 μm filters”.

A correction has been made to **Materials and methods**, *2.8.3 Sodium dodecyl sulfate-polyacrylamide gel electrophoresis*, Line 1. This sentence previously stated, “Twenty grams of proteins”. The corrected sentence is “Twenty micrograms of proteins”.

A correction has been made to **Materials and methods**, *2.8.4 Filter-aided sample preparation*, Line 11. This sentence previously stated, “NH_4_HCO_3_ buffer (40 mL)”. The corrected sentence is “NH_4_HCO_3_ buffer (40 μL)”.

A correction has been made to **Materials and methods**, *2.8.5 Mass spectrometry analysis*, Lines 4-5. This sentence previously stated, “an analytical column (25 cm×75 cm) containing beads (1.6 mm C18)”. The corrected sentence is “an analytical column (25 cm×75 μm) containing beads (1.6 μm C18)”.

A correction has been made to **Materials and methods**, *2.8.5 Mass spectrometry analysis*, Line 15. This sentence previously stated, “starting at 0.75 V/cm^2^ and ending at 1.4 V/cm^2^”. The corrected sentence is “starting at 0.75 V.s/cm^2^ and ending at 1.4 V.s/cm^2^ (1/K0)”.

The authors apologize for these errors and state that these do not change the scientific conclusions of the article in any way. The original article has been updated.

